# Temporal trends and rural–urban disparities in cerebrovascular risk factors, in-hospital management and outcomes in ischaemic strokes in China from 2005 to 2015: a nationwide serial cross-sectional survey

**DOI:** 10.1136/svn-2022-001552

**Published:** 2022-08-19

**Authors:** Chun-Juan Wang, Hong-Qiu Gu, Xin-Miao Zhang, Yong Jiang, Hao Li, Janet Prvu Bettger, Xia Meng, Ke-Hui Dong, Run-Qi Wangqin, Xin Yang, Meng Wang, Chelsea Liu, Li-Ping Liu, Bei-Sha Tang, Guo-Zhong Li, Yu-Ming Xu, Zhi-Yi He, Yi Yang, Winnie Yip, Gregg C Fonarow, Lee H Schwamm, Ying Xian, Xing-Quan Zhao, Yi-Long Wang, Yongjun Wang, Zixiao Li

**Affiliations:** 1 China National Clinical Research Center for Neurological Diseases, Beijing Tiantan Hospital, Capital Medical University, Beijing, China; 2 National Center for Healthcare Quality Management in Neurological Diseases, Beijing Tiantan Hospital, Capital Medical University, Beijing, China; 3 Vascular Neurology, Department of Neurology, Beijing Tiantan Hospital, Capital Medical University, Beiing, China; 4 Center of Stroke, Beijing Institute for Brain Disorders, Beijng, China; 5 Beijing Key Laboratory of Translational Medicine for Cerebrovascular Disease, Beijing, China; 6 Duke Clinical Research Institute, Duke University, Durham, North Carolina, USA; 7 Department of Neurology, Duke Univeristy Medical Center, Durham, North Carolina, USA; 8 Department of Epidemiology, Harvard T H Chan School of Public Health, Boston, Massachusetts, USA; 9 Neuro-intensive Care Unit, Department of Neurology, Beijing Tiantan Hospital, Capital Medical University, Beijing, China; 10 Department of Neurology, Xiangya Hospital, Central South University, Changsha, Hunan, China; 11 Department of Neurology, The First Affiliated Hospital of Harbin Medical University, Harbin, Heilongjiang, China; 12 Department of Neurology, The First Affiliated Hospital of Zhengzhou University, Zhengzhou, Henan, China; 13 Key Laboratory of Cerebrovascular Disease Prevention and Treatment, National Health Commission (Province and Ministry Co-constructed), Zhengzhou, Henan, China; 14 Department of Neurology, The First Affiliated Hospital of China Medical University, Shenyang, Liaoning, China; 15 Department of Neurology, The First Hospital of Jilin University, Changchun, Jilin, China; 16 Department of Global Health and Population, Harvard T. H. Chan School of Public Health, Boston, MA, USA; 17 Ahmanson-UCLA Cardiomyopathy Center, Ronald Reagan-UCLA Medical Center, Los Angeles, CA, USA; 18 Department of Neurology, Massachusetts General Hospital, Harvard Medical School, Boston, MA, USA; 19 Department of Neurology, The University of Texas Southwestern Medical Center, Dallas, Texas, USA; 20 Research Unit of Artificial Intelligence in Cerebrovascular Disease, Chinese Academy of Medical Sciences, Beijing, China; 21 Chinese Institute for Brain Research, Beijing, China

**Keywords:** Stroke

## Abstract

**Background:**

Stroke is the leading cause of mortality in China, with limited evidence of in-hospital burden obtained from nationwide surveys. We aimed to monitor and track the temporal trends and rural–urban disparities in cerebrovascular risk factors, management and outcomes from 2005 to 2015.

**Methods:**

We used a two-stage random sampling survey to create a nationally representative sample of patients admitted for ischaemic stroke in 2005, 2010 and 2015. We sampled participating hospitals with an economic-geographical region-stratified random-sampling approach first and then obtained patients with a systematic sampling approach. We weighed our survey data to estimate the national-level results and assess changes from 2005 to 2015.

**Results:**

We analysed 28 277 ischaemic stroke admissions from 189 participating hospitals. From 2005 to 2015, the estimated national hospital admission rate for ischaemic stroke per 100 000 people increased (from 75.9 to 402.7, P_trend_<0.001), and the prevalence of risk factors, including hypertension, diabetes, dyslipidaemia and current smoking, increased. The composite score of diagnostic tests for stroke aetiology assessment (from 0.22 to 0.36, P_trend_<0.001) and secondary prevention treatments (from 0.46 to 0.70, P_trend_<0.001) were improved. A temporal decrease was found in discharge against medical advice (DAMA) (from 15.2% (95% CI 13.7% to 16.7%) to 8.6% (8.1% to 9.0%); adjusted P_trend_=0.046), and decreases in in-hospital mortality (0.7% in 2015 vs 1.8% in 2005; adjusted OR (aOR) 0.52; 95% CI 0.32 to 0.85) and the composite outcome of in-hospital mortality or DAMA (8.4% in 2015 vs 13.9% in 2005; aOR 0.65; 95% CI 0.47 to 0.89) were observed. Disparities between rural and urban hospitals narrowed; however, disparities persisted in in-hospital management (brain MRI: rural–urban difference from −14.4% to −11.2%; cerebrovascular assessment: from −20.3% to −16.7%; clopidogrel: from −2.1% to −10.3%; anticoagulant for atrial fibrillation: from −10.9% to −8.2%) and in-hospital outcomes (DAMA: from 2.7% to 5.0%; composite outcome of in-hospital mortality or DAMA: from 2.4% to 4.6%).

**Conclusions:**

From 2005 to 2015, improvements in hospital admission and in-hospital management for ischaemic stroke in China were found. A temporal improvement in DAMA and improvements in in-hospital mortality and the composite outcome of in-hospital mortality or DAMA were observed. Disparities between rural and urban hospitals generally narrowed but persisted.

**WHAT IS ALREADY KNOWN ON THIS TOPIC**

- Stroke burden in China has risen over the past three decades, with significant urban–rural variations across the country. However, previous evidence was not obtained from nationwide surveys.

**WHAT THIS STUDY ADDS**

- In this nationwide serial cross-sectional survey, increases in the prevalence of some cerebrovascular risk factors were found. Improvements in in-hospital management and outcomes were observed, and disparities between rural and urban hospitals generally narrowed but persisted.

**HOW THIS STUDY MIGHT AFFECT RESEARCH, PRACTICE OR POLICY**

- These findings underscore the need for tailored strategies for resource allocation and disease management in the coming decades.

## Introduction

China has concurrently experienced epidemiological and demographic transitions along with rapid economic growth in recent decades.^1 2^ The national stroke burden has risen over the past three decades, with a higher number of prevalent stroke cases than in other countries and significant urban–rural variations across the country.^3–6^ To respond to these challenges, the Chinese government has implemented policies since 2009 to provide affordable and equitable healthcare for all citizens by 2020. These policies involve increasing medical insurance coverage, improving medical care and providing essential public health services.^7 8^

Although ischaemic stroke accounts for more than 80% of stroke events in China,^9 10^ previous studies on trends in the management and outcomes of inpatients with ischaemic stroke were largely limited to hospitals in urban regions,^11^ a small subset of provinces,^12^ or the timeline did not cover the years of China’s healthcare reform.^9^ Therefore, a nationally representative study across the years of China’s healthcare reform is needed to provide high-level evidence for the development of tailored strategies for resource allocation and stroke management for the coming decades.

In the Patient-centered Retrospective Observation of Guideline-Recommended pErformance for Stroke Sufferers (China PROGRESS) Study, we aimed to address these gaps in knowledge to provide up-to-date real-world evidence of national temporal trends in quality of care and outcomes for patients hospitalised for ischaemic stroke between 2005 and 2015.

## Methods

### Study design

Details on the study design for China PROGRESS have been described elsewhere.^13^ Briefly, we conducted a two-stage random sampling design to create a nationally representative sample of patients admitted for ischaemic stroke in China in 2005, 2010 and 2015 using a sampling framework similar to the China PEACE (Patient-centered Evaluative Assessment of Cardiac Events) Study.^14^

In the first stage, we excluded military, prison, specialised and traditional Chinese medicine hospitals. All remaining hospitals were stratified into five economic-geographical regions: eastern rural, central rural, western rural, eastern urban and central-western urban. The central and western urban areas were grouped into one stratum due to similar income levels and per capita health service capacities.^14^ ^15^ All secondary and tertiary hospitals in urban regions and all central hospitals in rural regions were randomly sampled. Last, hospitals that did not admit patients with ischaemic stroke or declined to participate or provide sample cases were excluded.

In the second stage, we used systematic random sampling procedures to select patients hospitalised for ischaemic stroke from each hospital’s database in 2005, 2010 and 2015. Patients with ischaemic stroke were identified through medical records by principal diagnosis at discharge using version 10 of the International Classification of Disease (ICD-10) Clinical Modification codes (I63.x). All sampled medical records were scanned through a high-speed scanner by trained researchers and transmitted to the National Coordinating Center through an encrypted, predesigned pathway. The process of scanning and transmission was regularly monitored and audited, and double-blinded data reading and entry of the medical record copies were performed. Two independent personnel were trained to extract data from medical record copies. A double-check data entry system was used for quality control, followed by a third party recheck for inconsistent entries.^13^

### Procedures

Age, sex, stroke risk factors, laboratory tests, and other clinical characteristics of patients who were hospitalised and discharged for ischaemic stroke were extracted from the medical record copies by trained personnel. Comorbidities, including hypertension, diabetes, atrial fibrillation and dyslipidaemia, were recorded by checking admission notes or discharge diagnoses. The information on diagnostic testing for stroke aetiology included CT, MRI scan, cerebrovascular assessment (including brain CT angiography (CTA), magnetic resonance angiography (MRA), transcranial Doppler and/or digital subtraction angiography (DSA)), cervical vessel assessment (including cervical CTA, MRA, carotid ultrasound and/or DSA), cardiac ultrasound (including transthoracic echocardiography (TTE) and transoesophageal echocardiography (TOE)), Holter ECG monitoring and 24-hour blood pressure monitoring; and in-hospital treatments, including antiplatelets, antihypertension and anticoagulation, were all extracted by checking related documentation.

### Study variables for management and outcomes

The primary outcome was all-cause in-hospital mortality. The secondary outcomes included discharge against medical advice (DAMA), a composite outcome of in-hospital death and DAMA, complications, length of stay (LOS) and cost. We also assessed adherence to diagnostic tests and secondary prevention treatments during hospitalisation.

Two composite scores were developed for each patient to summarise adherence to guideline-directed diagnostic tests or secondary prevention treatments. The composite score was defined as the total number of tests or treatments performed among eligible patients divided by the total number of possible tests or treatments among eligible patients.^16^ We used the China National Guidelines for Stroke and Transient Ischaemic Attack (TIA) Management (2014) for definitions of adherence to treatment guidelines; some of the measures are consistent with the guideline-based recommendations adopted by ‘Get With The Guidelines-Stroke’ for measuring adherence in the USA.^16 17^ These measures include diagnostic tests for stroke aetiology, acute management, medications for secondary prevention (antiplatelet (aspirin and clopidogrel), anticoagulants for atrial fibrillation, statins, antihypertensives, antidiabetic medication), dysphagia screening and rehabilitation assessment in patients without documented contraindications. Traditional Chinese medicine use was assessed separately due to a lack of solid evidence of its efficacy for stroke treatment.^18^ ^19^

Detailed definitions or specifications of outcome measures or performance indicators are shown in online supplemental tables 1 and 2. Patients who were transferred to another hospital were excluded from the analysis of in-hospital mortality because there was very limited time to capture in-hospital death. Patients who were discharged against medical advice but not transferred to another hospital or rehabilitation centre may indicate terminal status and willingness to die at home due to culture or affordability.^14^ Therefore, we excluded patients who died during hospitalisation (impossible to DAMA), were transferred to another hospital or a rehabilitation centre (may seek further treatments) or had no documentation of detailed destination (missing values) from the analysis of DAMA for eligibility. In addition, we combined the in-hospital mortality and DAMA as a composite outcome because it is common (approximately 16%) for patients who had a stroke to withdraw from treatment at unfavourable or terminal status in China,^10^ and this may represent a unique discharge pattern in China as well.

10.1136/svn-2022-001552.supp1 Supplementary data

### Statistical analyses

To estimate the national-level results, we applied weights proportional to the inverse sampling fraction of hospitals within each stratum in the first stage and the inverse sampling fraction of patients within each hospital in the second stage. We calculated the rate of hospital admissions due to ischaemic stroke each year by dividing the projected number of admitted patients by the total number of the adult population in China obtained from the National Bureau of Statistics (1.04 billion in 2005, 1.11 billion in 2010 and 1.16 billion in 2015). Unweighted frequencies and weighted percentages along with 95% CIs were used to describe the categorical variables; weighted means along with SDs (mean±SD) or medians along with IQRs (median (IQR)) were used to describe continuous variables. We examined trends across the 3 study years using the Cochran-Armitage trend test for binary variables, the Cochran-Mantel-Haenszel (CMH) row-mean score test for categorical variables with three or more levels, and the CMH non-zero correlation test for continuous variables.

Logistic regression models with generalised estimating equations were used to account for within-hospital clustering for the assessment of in-hospital outcomes (in-hospital mortality, DAMA, in-hospital mortality or DAMA). Based on a comprehensive consideration of statistical perspectives and a literature review, models were adjusted for age, sex, insurance type (Urban Employee Basic Medical Insurance; Urban Resident Basic Medical Insurance; New Rural Co-operative Medical Insurance Scheme, self-payment or other),^20^ and history of hypertension, diabetes mellitus, dyslipidaemia, atrial fibrillation, smoking, stroke, coronary heart disease or myocardial infarction. All covariates had complete data except for age (<1% missing), smoking history (<3% missing) and stroke history (<3% missing) (see online supplemental table 3 for details). Missing entries for age were imputed to median age, while missing entries for smoking history and stroke history were imputed to having no history. We treated year as a continuous variable in the adjusted models to estimate p values for trend analysis of in-hospital outcomes. Analyses for diagnostic tests, in-hospital treatments and comorbidities were not adjusted for covariates, and the results were exploratory.

To estimate discrepancies between rural and urban areas, we estimated risk differences for categorical variables and Hodges-Lehmann estimation of location shift for continuous variables along with their 95% CIs by year. P values for the interaction items of location and year in logistic regression models were also estimated to assess the trends in urban–rural discrepancies by year. We also reported adjusted ORs (aORs) and 95% CIs derived from logistic regression models with generalised estimating equations to account for the within-hospital clustering effect.

All comparisons were two sided, with statistical significance defined as p<0.05. All analyses were performed using SAS V.9.4 software (SAS Institute).

## Results

**Figure 1:**
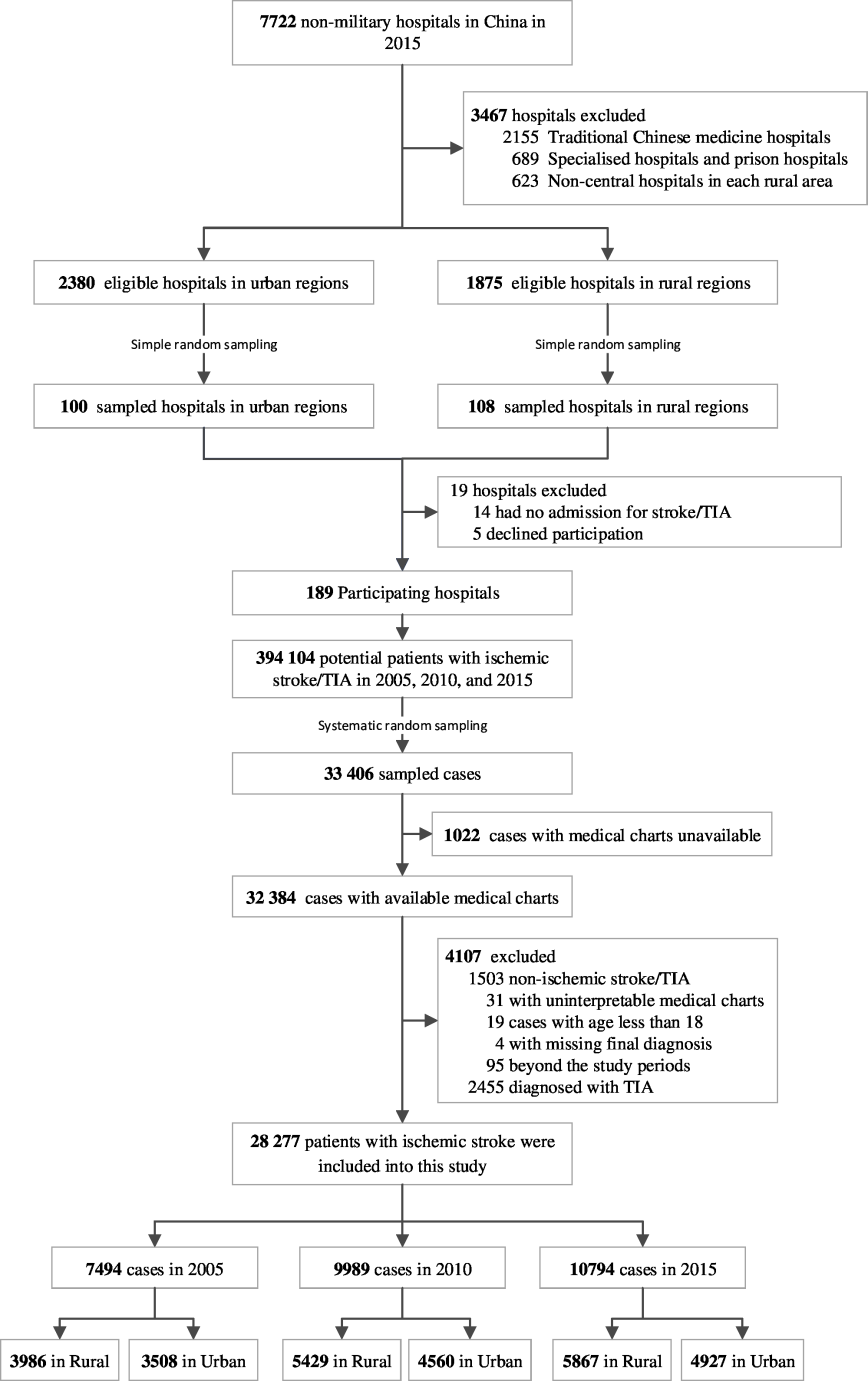
Study profile. TIA, transient ischaemic attack.

**Figure 2:**
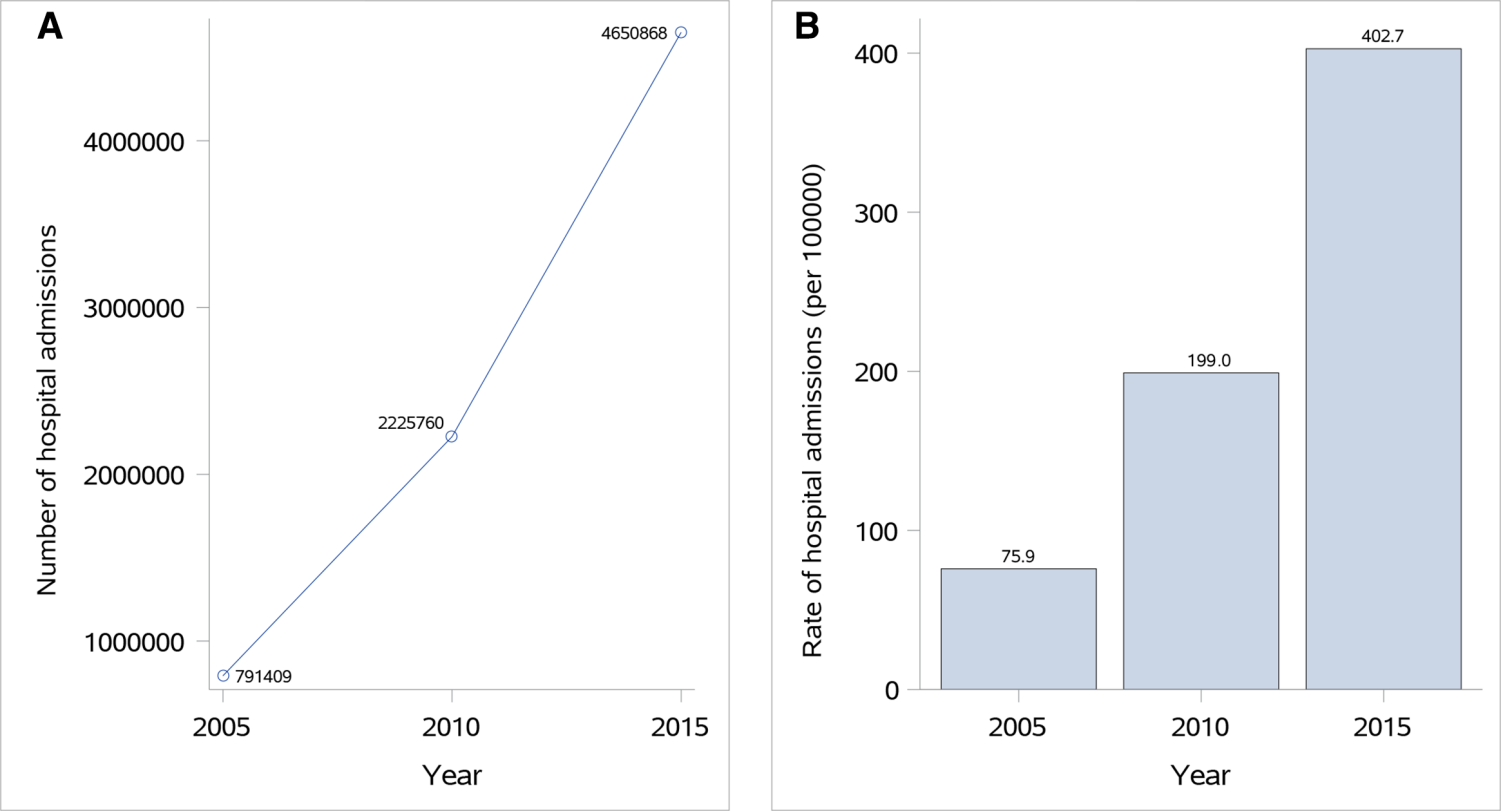
Hospital admissions for ischaemic stroke in China in 2005, 2010 and 2015: (A) number and (B) rate (per 100 000) of hospital admissions for ischaemic stroke.

The sampling framework consisted of 1875 central hospitals in 1875 rural areas across three rural strata and 2380 secondary or tertiary hospitals in 295 urban regions across two urban strata. Among them, we sampled a total of 208 hospitals and invited them to participate in this study. Of the 19 hospitals that did not participate in this study, 14 of them did not admit patients with ischaemic stroke, and 5 of them declined the invitation. There were 394 104 hospital admissions for ischaemic stroke or TIA (41 591 in 2005, 113 592 in 2010 and 238 921 in 2015) among the patient databases from the 189 participating hospitals, of which we sampled 33 406 for this study. After excluding 1022 cases with unavailable medical charts and 4107 cases that did not meet other study criteria, the final study sample consisted of 28 277 patients admitted for ischaemic stroke (7494 from 2005, 9989 from 2010 and 10 794 from 2015) (figure 1). Based on these data, we estimated the number of patients admitted for ischaemic stroke in China nationwide to be 791 409 in 2005, 2 225 760 in 2010 and 4 650 868 in 2015. The rate of hospital admission for ischaemic stroke per 100 000 people was 75.9 in 2005, 199.0 in 2010 and 402.7 in 2015 (P_trend_<0.001) (figure 2).

### Cerebrovascular risk factors

From 2005 to 2015, the female percentage of patients admitted for ischaemic stroke did not change significantly (from 38.7% to 39.8%, table 1). The prevalence of all cerebrovascular risk factors and previous atherosclerotic events increased over time, with the exception of atrial fibrillation and myocardial infarction. The proportion of patients without health insurance (ie, self-payment) decreased over time (from 39.4% to 8.2%, P_trend_<0.001; table 1). The median time from symptom onset to hospital admission remained 2 days during the three periods, but the percentage of patients who were admitted over 8 days after onset increased over time (from 17.2% in 2005 to 20.1% in 2015, P_trend_<0.001; table 1). Between 2005 and 2015, patients became less likely to present with coma, limb weakness, aphasia or dysarthria. The mean values of systolic blood pressure, median cholesterol, low-density lipoproteins and alanine transaminase decreased. In contrast, the median fasting glucose, uric acid concentration and estimated glomerular filtration rate significantly increased (table 1).

**Table 1:** Demographic characteristics of patients stratified by years

	2005 (N=7494 (26.5%))	2010 (N=9989 (35.3%))	2015 (N=10 794 (38.2%))	P_trend_
**Demographic**				
Age, year (median, IQR)	67.0 (57.0–74.0)	68.0 (59.0–75.0)	67.0 (59.0–76.0)	<0.001
Women (no (weighted %, 95% CI))	3001 (38.7 (36.9 to 40.5))	4069 (40.2 (39.1 to 41.3))	4516 (39.8 (39.0 to 40.5))	0.60
**Cerebrovascular risk factors (no (weighted %, 95% CI)**)				
Hypertension	5310 (70.5 (68.8 to 72.2))	7474 (76.7 (75.8 to 77.6))	7961 (74.8 (74.2 to 75.5))	0.02
Diabetes	1466 (22.4 (20.9 to 23.9))	2072 (25.1 (24.1 to 26.0))	2545 (27.8 (27.1 to 28.5))	<0.001
Dyslipidaemia	567 (7.4 (6.4 to 8.4))	1328 (14.7 (13.9 to 15.4))	2109 (23.3 (22.7 to 23.9))	<0.001
Atrial fibrillation	514 (6.6 (5.7 to 7.5))	582 (5.1 (4.6 to 5.6))	696 (5.6 (5.3 to 6.0))	0.47
Current smoker	1024 (13.9 (12.6 to 15.1))	1550 (17.5 (16.7 to 18.4))	1581 (19.4 (18.7 to 20.0))	<0.001
Number of risk factors ≥3	552 (7.7 (6.7 to 8.6))	1479 (17.1 (16.3 to 17.9))	2544 (27.5 (26.8 to 28.1))	<0.001
**Medical history (no (weighted %, 95% CI)**)				
Stroke	1351 (21.2 (19.7 to 22.7))	2166 (23.1 (22.2 to 24.1))	2738 (29.2 (28.5 to 29.9))	<0.001
Coronary heart disease	1446 (21.0 (19.5 to 22.5))	2009 (23.6 (22.7 to 24.5))	2570 (27.0 (26.3 to 27.7))	<0.001
Myocardial infarction	112 (1.6 (1.2 to 2.1))	131 (1.4 (1.2 to 1.7))	175 (1.9 (1.6 to 2.1))	0.07
**Insurance status (no (weighted %, 95% CI)**)				
Urban Employee Basic Medical Insurance	1420 (24.6 (23.0 to 26.1))	2254 (30.4 (29.4 to 31.4))	3186 (33.6 (32.9 to 34.3))	<0.001
Urban Resident Basic Medical Insurance	0	811 (10.7 (10.0 to 11.4))	1010 (9.3 (8.9 to 9.8))	
New Rural Co-operative Medical Insurance Scheme	142 (1.6 (1.1 to 2.0))	2234 (18.9 (18.0 to 19.7))	4275 (33.4 (32.7 to 34.1))	
Self-payment	3395 (39.4 (37.6 to 41.2))	1583 (15.8 (15.0 to 16.6))	914 (8.2 (7.8 to 8.6))	
Other	2537 (34.5 (32.7 to 36.2))	3107 (24.1 (23.2 to 25.1))	1409 (15.5 (14.9 to 16.0))	
**Region (no (weighted %, 95% CI)**)				
Rural	3986 (25.6 (24.0 to 27.2))	5429 (32.0 (31.0 to 33.0))	5867 (34.7 (34.0 to 35.4))	<0.001
Urban	3508 (74.4 (72.8 to 76.0))	4560 (68.0 (67.0 to 69.0))	4927 (65.3 (64.6 to 66.0))	
**Clinical characteristic**				
Time from symptom onset to admission (days; median (IQR))	2.0 (0.0–5.0)	2.0 (0.0–6.0)	2.0 (0.0–7.0)	<0.001
Time from symptom onset to admission (days; no (weighted %, 95% CI))				
<1	2491 (30.7 (29.1 to 32.4))	2906 (27.7 (26.7 to 28.6))	2873 (26.4 (25.8 to 27.1))	
1–7	3858 (52.1 (50.3 to 53.9))	5259 (54.2 (53.1 to 55.3))	5825 (53.5 (52.7 to 54.2))	
≥8	1138 (17.2 (15.8 to 18.5))	1814 (18.2 (17.3 to 19.0))	2088 (20.1 (19.5 to 20.7))	
Coma (no (weighted %, 95% CI))	214 (2.3 (1.7 to 2.8))	175 (1.6 (1.3 to 1.9))	158 (1.6 (1.4 to 1.8))	0.044
Limb weakness (muscle power 0–4) (no (weighted %, 95% CI))	3089 (38.4 (36.6 to 40.2))	3633 (34.6 (33.6 to 35.6))	3391 (33.2 (32.5 to 34.0))	<0.001
Limb weakness (muscle power 0–3) (no (weighted %, 95% CI))	1984 (24.0 (22.4 to 25.5))	2179 (19.5 (18.6 to 20.3))	1873 (17.4 (16.9 to 18.0))	<0.001
Aphasia or dysarthria (no (weighted %, 95% CI))	2031 (29.0 (27.4 to 30.7))	2428 (28.8 (27.8 to 29.8))	2533 (27.3 (26.6 to 27.9))	0.007
Systolic blood pressure (mm Hg; mean (SD))	150.2±16.2	148.3±22.1	145.6±28.8	<0.001
Cholesterol (mmol/L; median (IQR))	4.8 (4.1–5.5)	4.7 (4.0–5.5)	4.6 (3.8–5.3)	<0.001
Low-density lipoprotein (mmol/L; median (IQR))	2.9 (2.3–3.4)	2.8 (2.2–3.4)	2.6 (2.1–3.3)	<0.001
Fast glucose (mmol/L; median (IQR))	5.5 (4.8–6.8)	5.5 (4.8–6.7)	5.6 (4.9–6.9)	0.022
Uric acid (μmol/L; median (IQR))	291.0 (227.0–364.0)	294.0 (237.9–363.0)	308.2 (248.0–376.9)	<0.001
Alanine transaminase (U/L; median (IQR))	18.5 (14.0–28.0)	18.0 (13.0–26.0)	18.0 (13.0–26.0)	<0.001
Estimated glomerular filtration rate (mL/(min/1.73 m^2^))	83.0 (65.7–100.0)	91.4 (72.0–104.6)	95.4 (78.6–107.3)	<0.001
≥90	2000 (40.4 (38.4 to 42.4))	4186 (52.4 (51.3 to 53.6))	5557 (60.4 (59.6 to 61.2))	<0.001
<90	3386 (59.6 (57.6 to 61.6))	4392 (47.6 (46.4 to 48.7))	4087 (39.6 (38.8 to 40.4))	<0.001

Data are presented by weighted mean along with SD (mean±SD) or median along with IQR (median (IQR)) for continuous variables, and unweighted frequency along with weighted % (95% CI) for categorical variables.

### Diagnostic tests and evidence-based treatments

The proportion of those who underwent tests for CT/MRI (from 80.8% to 84.6%), cerebrovascular assessment (from 20.4% to 41.8%), cervical vessel assessment (from 13.6% to 32.4%), cardiac ultrasound (TTE or TOE) (from 13.9% to 36.9%), Holter ECG monitoring (from 6.0% to 11.6%), and 24-hour blood pressure monitoring (from 3.2% to 8.6%) increased between 2005 and 2015 (all P_trend_<0.001, table 2). Over time, the composite score of diagnostic tests for stroke aetiology assessment significantly increased (from 0.22±0.11 to 0.36±0.28, P_trend_<0.001; table 2).

**Table 2:** Diagnostic tests and in-hospital treatments stratified by years

Tests or treatments	2005 (N=7028 (26.1%))		2010 (N=9498 (35.3%))		2015 (N=10 374 (38.6%))		P_trend_
	Relative frequency	Weighted % (95% CI)	Relative frequency	Weighted % (95% CI)	Relative frequency	Weighted % (95% CI)	
Diagnostic tests							
Brain CT/MRI	5334/6777	80.8 (79.3 to 82.3)	7495/9192	83.6 (82.8 to 84.4)	8392/10 024	84.6 (84.0 to 85.2)	<0.001
Brain CT	4922/6776	71.1 (69.4 to 72.8)	6483/9191	68.3 (67.2 to 69.3)	6887/10 023	64.2 (63.5 to 65.0)	<0.001
Brain MRI	923/6777	24.2 (22.6 to 25.8)	2448/9192	38.9 (37.8 to 40.0)	3699/10 023	47.3 (46.5 to 48.1)	<0.001
Cerebrovascular assessment*	729/6978	20.4 (18.9 to 21.9)	1970/9469	33.7 (32.6 to 34.7)	3399/10 322	41.8 (41.1 to 42.6)	<0.001
Cervical vessel assessment†	405/7028	13.6 (12.3 to 14.9)	1419/9498	21.8 (20.9 to 22.7)	2792/10 374	32.4 (31.7 to 33.1)	<0.001
TTE/TOE	1016/6778	13.9 (12.6 to 15.2)	2148/9192	28.0 (27.0 to 29.1)	3543/10 023	36.9 (36.2 to 37.7)	<0.001
TTE	1006/6777	13.8 (12.4 to 15.1)	2116/9192	27.8 (26.8 to 28.8)	3486/10 023	36.4 (35.7 to 37.2)	<0.001
TEE	23/6778	0.3 (0.1 to 0.5)	163/9189	1.9 (1.6 to 2.2)	344/10 023	3.9 (3.6 to 4.2)	<0.001
Holter	355/6777	6.0 (5.0 to 6.9)	873/9187	9.6 (8.9 to 10.2)	1053/10 020	11.6 (11.1 to 12.1)	<0.001
24-hour blood pressure monitor	199/6777	3.2 (2.5 to 3.9)	496/9187	4.6 (4.2 to 5.1)	743/10 023	8.6 (8.2 to 9.1)	<0.001
Composite score of diagnostic tests (mean±SD)‡	0.22±0.11		0.30±0.19		0.36±0.28		<0.001
Homocysteine	36/7028	0.6 (0.3 to 0.9)	855/9498	17.3 (16.4 to 18.1)	3726/10 374	42.5 (41.7 to 43.2)	<0.001
Glycated haemoglobin	229/7028	4.1 (3.3 to 4.8)	1252/9498	20.0 (19.1 to 20.9)	3150/10 374	34.5 (33.8 to 35.2)	<0.001
rt-PA use when arrived within 1 day after onset§	16/3579	0.2 (0.0 to 0.5)	2/4374	0.1 (0.0 to 0.1)	39/4579	1.4 (1.1 to 1.6)	<0.001
Secondary prevention treatments							
Antiplatelet	4457/7011	60.5 (58.7 to 62.4)	6966/9490	75.9 (75.0 to 76.9)	8124/10 354	78.6 (78.0 to 79.3)	<0.001
Aspirin	4302/7011	57.3 (55.4 to 59.1)	6622/9490	68.1 (67.1 to 69.1)	7229/10 354	67.8 (67.1 to 68.5)	<0.001
Clopidogrel	67/7011	2.2 (1.6 to 2.7)	692/9490	13.4 (12.6 to 14.2)	2410/10 354	29.3 (28.6 to 30.0)	<0.001
Aspirin plus clopidogrel	40/7011	1.1 (0.7 to 1.5)	337/9490	5.3 (4.8 to 5.8)	1478/10 354	17.9 (17.3 to 18.5)	<0.001
Anticoagulant for atrial fibrillation	21/461	9.9 (5.5 to 14.4)	32/537	8.1 (5.4 to 10.8)	62/649	13.3 (11.0 to 15.5)	0.030
Antihypertensive drugs	3686/5031	72.4 (70.5 to 74.4)	5150/7140	68.7 (67.5 to 69.8)	5132/7670	64.9 (64.1 to 65.8)	<0.001
Hypoglycaemic drugs	968/1400	66.3 (62.6 to 69.9)	1460/1990	74.3 (72.3 to 76.2)	1736/2453	69.5 (68.2 to 70.8)	0.42
Statins	885/6912	14.4 (13.1 to 15.7)	3599/9283	42.9 (41.7 to 44.0)	6727/9858	71.6 (70.9 to 72.3)	<0.001
Composite score of secondary prevention treatments¶	0.46±0.18		0.61±0.29		0.70±0.41		<0.001
Traditional Chinese medications	5500/7028	73.7 (72.1 to 75.4)	8297/9498	86.3 (85.5 to 87.0)	8716/10 374	83.2 (82.6 to 83.8)	<0.001
Intervention							
Dysphagia screening	180/7028	3.3 (2.7 to 4.0)	472/9497	6.7 (6.2 to 7.3)	683/10 373	9.7 (9.2 to 10.1)	<0.001
Rehabilitation assessment	364/6561	7.6 (6.6 to 8.6)	656/9019	7.8 (7.2 to 8.4)	847/9915	8.7 (8.3 to 9.2)	0.007

*Includes brain CT angiography, magnetic resonance angiography, transcranial Doppler and/or digital subtraction angiography.

†Included cervical CT angiography, magnetic resonance angiography, carotid ultrasound and/or digital subtraction angiography.

‡Composite score of tests was derived from brain CT/MRI, cerebrovascular assessment, cervical vessel assessment, TTE/TOE, Holter and 24-hour blood pressure monitor.

§For most patients, the onset time and admission time to hospitals were not documented, and we could only calculate the time intervals by day.

¶Composite score of secondary prevention treatments was derived from in-hospital antiplatelets, anticoagulant for atrial fibrillation, antihypertensive drugs, hypoglycaemic drugs and statins therapy.

rt-PA, recombinant tissue plasminogen activator; TEE, transoesophageal echocardiography; TTE, transthoracic echocardiography.

The rate of recombinant tissue plasminogen activator (rt-PA) utilisation increased slightly in patients with ischaemic stroke who arrived within 1 day after onset over the 10 years (from 0.2% to 1.4%, P_trend_<0.001; table 2). The weighted proportion of patients receiving guideline-recommended stroke care increased between 2005 and 2015 for in-hospital secondary prevention treatments, including antiplatelets (from 60.5% to 78.6%, P_trend_<0.001; table 2), anticoagulation for atrial fibrillation (from 9.9% to 13.3%, P_trend_=0.030; table 2) and statins (from 14.4% to 71.6%, P_trend_<0.001; table 2). However, the prescription of hypoglycaemic drugs among eligible patients did not improve significantly (from 66.3% to 69.5%, P_trend_=0.423; table 2), and the prescription of antihypertensives decreased (from 72.4% to 64.9%, P_trend_<0.001; table 2). In general, the composite score of in-hospital secondary prevention treatments demonstrated a remarkable increase (from 0.46±0.18 to 0.70±0.41, P_trend_<0.001; table 2). In addition, the use of traditional Chinese medicine during hospitalisation increased (from 73.7% to 83.2%, P_trend_<0.001; table 2). The top 10 traditional Chinese medicines used each year are listed in online supplemental table 4.

### In-hospital outcomes

**Figure 3:**
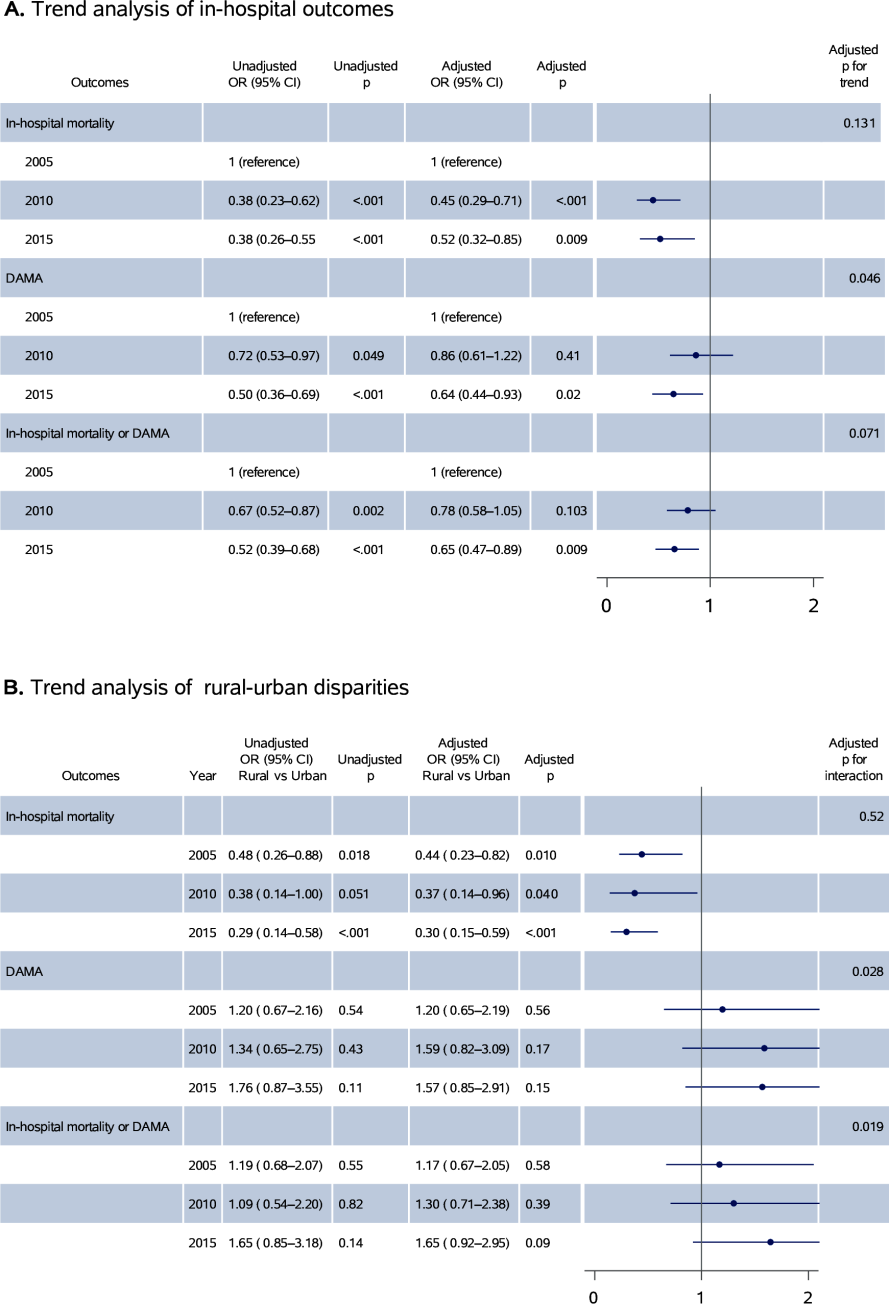
Trends of in-hospital outcomes for patients with ischaemic stroke from 2005 to 2015. (A) Overall trends in in-hospital outcomes for patients with ischaemic stroke; (B) trends of rural–urban disparities in in-hospital outcomes for patients with ischaemic stroke. DAMA, discharge against medical advice.

A total of 133 out of 7324 patients died during hospitalisation in 2005 (unadjusted weighted mortality rate 1.8%, 95% CI 1.3% to 2.3%); 74 out of 9796 patients died in 2010 (0.7%, 95% CI 0.5% to 0.9%) and 67 out of 10 538 died in 2015 (0.7%, 95% CI 0.6% to 0.8%). A temporal trend was seen in the crude analysis (P_trend_<0.001). The rate of DAMA (15.2% in 2005, 11.8% in 2010 and 8.6% in 2015) and the composite outcome of in-hospital mortality or DAMA (13.9% in 2005, 9.9% in 2010 and 8.4% in 2015) also decreased significantly over time (P_trend_<0.001; table 3). After adjustment for patients’ demographic and clinical characteristics, a significant temporal decrease was found in DAMA (P_trend_=0.046) but not in in-hospital mortality (P_trend_=0.131) or for the composite outcome of in-hospital mortality or DAMA (P_trend_=0.071) from 2005 to 2015. However, compared with 2005, the odds of in-hospital mortality were significantly lower in 2010 (aOR 0.45; 95% CI 0.29 to 0.71) and 2015 (aOR 0.52; 95% CI 0.32 to 0.85), and the odds of DAMA (aOR 0.64; 95% CI 0.44 to 0.93) and the composite outcome of in-hospital mortality or DAMA (aOR 0.65; 95% CI 0.47 to 0.89) were also significantly lower in 2015 (figure 3A).

**Table 3:** In-hospital outcomes, comorbidities and costs stratified by years

	2005 (N=7494 (26.5%))		2010 (N=9989 (35.3%))		2015 (N=10 794 (38.2%))		P_trend_
	Relative frequency	Weighted % (95% CI)	Relative frequency	Weighted % (95% CI)	Relative frequency	Weighted % (95% CI)	
In-hospital outcomes							
In-hospital mortality*	133/7324	1.8 (1.3 to 2.3)	74/9796	0.7 (0.5 to 0.9)	67/10 538	0.7 (0.6 to 0.8)	<0.001
DAMA†	1205/6252	15.2 (13.7 to 16.7)	1254/8257	11.8 (11.0 to 12.6)	1069/9710	8.6 (8.1 to 9.0)	<0.001
In-hospital mortality or DAMA‡	1338/7292	13.9 (12.6 to 15.2)	1328/9757	9.9 (9.3 to 10.6)	1136/10 473	8.4 (8.0 to 8.8)	<0.001
Comorbidities							
Pneumonia	700/7494	9.8 (8.7 to 10.9)	999/9989	9.4 (8.8 to 10.0)	1204/10 794	10.4 (10.0 to 10.9)	0.039
Heart failure	323/7494	3.4 (2.7 to 4.1)	482/9989	3.9 (3.5 to 4.3)	708/10 794	5.5 (5.2 to 5.9)	<0.001
Cerebral haemorrhage	115/7494	1.9 (1.4 to 2.4)	102/9989	0.9 (0.7 to 1.1)	168/10 794	1.4 (1.2 to 1.6)	0.81
Symptomatic seizure	90/7494	1.3 (0.9 to 1.7)	114/9989	1.4 (1.2 to 1.7)	98/10 794	1.0 (0.8 to 1.1)	0.007
Depression	12/7494	0.5 (0.2 to 0.7)	31/9989	0.4 (0.3 to 0.5)	47/10 794	0.6 (0.5 to 0.8)	0.060
Deep vein thrombosis	15/7494	0.4 (0.2 to 0.6)	15/9989	0.2 (0.1 to 0.3)	49/10 794	0.4 (0.3 to 0.5)	0.30
Renal dysfunction	125/7494	1.7 (1.2 to 2.2)	211/9989	2.4 (2.1 to 2.8)	388/10 794	3.5 (3.2 to 3.8)	<0.001
Liver dysfunction	116/7494	1.4 (1.0 to 1.9)	220/9989	2.3 (2.0 to 2.6)	524/10 794	4.9 (4.6 to 5.2)	<0.001
LOS and costs*							
LOS (days)	13.0 (7.0–18.0)		13.0 (8.0–15.0)		11.0 (8.0–14.0)		<0.001
Total costs (¥)§	4169.0 (2375.0–7130.0)		5406.8 (3077.8–8698.5)		5676.3 (3578.0–8767.1)		<0.001
Drug costs (¥)§	2642.2 (1465.9–4453.9)		3031.2 (1701.4–5138.5)		2852.8 (1551.3–4824.0)		0.18

Data are presented by weighted median along with IQR (median (IQR)) for continuous variables and unweighted frequency along with weighted % (95% CI) for categorical variables.

*A total of 619 patients (170, 193, and 256 in 2005, 2010, and 2015, respectively) who were transferred to another hospital were all excluded from this analysis.

†A total of 4058 patients (1241, 1732, and 1084 in 2005, 2010, and 2015, respectively) who died during hospitalisation were transferred to another hospital or rehabilitation centre, or had no documentation of a detailed destination were all excluded from this analysis.

‡Seven hundred fifty-five patients (202, 232, and 321 in 2005, 2010, and 2015, respectively) who were transferred out to another hospital or a rehabilitation centre were all excluded from this analysis.

§Consumer price index-adjusted cost.

DAMA, discharge against medical advice; LOS, length of stay.

The proportion of patients who experienced comorbidities, including pneumonia (from 9.8% to 10.4%), heart failure (from 3.4% to 5.5%), renal dysfunction (from 1.7% to 3.5%) and liver dysfunction (from 1.4% to 4.9%) increased, but symptomatic seizures decreased (from 1.3% to 1.0%) between 2005 and 2015 (P_trend_<0.001, table 3). Although the median LOS decreased from 13 days to 11 days, the median total hospitalisation costs increased slightly over time (from ¥4169 to ¥5676), but drug costs remained unchanged (table 3).

### Temporal trends in rural–urban disparities

Disparities between rural and urban hospitals for the proportion of admitted female patients increased (from 1.1% to 6.9%), current smokers (from 2.5% to −8.5%), and patients with aphasia or dysarthria (from 0.0% to −7.4%) between 2005 and 2015 (online supplemental table 5). Disparities between rural and urban areas for some diagnostic tests and in-hospital treatments, including brain MRI (from −14.4% (−17.7% to – 11.1%) to −11.2% (−12.9% to −9.6%)), cerebrovascular assessment (from −20.3% (−22.8% to – 17.8%) to −16.7% (−18.3% to −15.2%)) and use of anticoagulant for atrial fibrillation (from −10.9% (−18.1% to – 3.8%) to −8.2% (−12.3% to −4.1%)) decreased; however, gaps still exist (table 4). Significant disparities between rural and urban areas were found for in-hospital mortality, and the disparities in mortality did not change significantly from 2005 to 2015 (aOR (95% CI), 0.44 (0.23 to 0.82) in 2005, 0.37 (0.14 to 0.96) in 2010 and 0.30 (0.15 to 0.59) in 2015; p for interaction=0.52); however, odds for DAMA (aOR (95% CI), 1.20 (0.65 to 2.19) in 2005, 1.59 (0.82 to 3.09) in 2010 and 1.57 (0.85 to 2.91) in 2015; p for interaction=0.028) and the composite outcome of in-hospital mortality or DAMA (aOR (95% CI), 1.17 (0.67 to 2.05) in 2005, 1.30 (0.71 to 2.38) in 2010 and 1.65 (0.92 to 2.95) in 2015; p for interaction=0.019) increased from 2005 to 2015 (table 5, figure 3B).

**Table 4:** Diagnostic tests and in-hospital treatments of all patients stratified by years and urban/rural

Tests or treatments	2005			2010			2015			P for interaction
	Rural (N=3680)	Urban (N=3348)	Difference % (95% CI)	Rural (N=5128)	Urban (N=4370)	Difference % (95% CI)	Rural (N=5619)	Urban (N=4755)	Difference % (95% CI)	
Diagnostic tests										
Brain CT/MRI	2795 (76.9)	2539 (82.1)	−5.3 (−8.9 to −1.6)	3964 (79.2)	3531 (85.6)	−6.4 (−8.3 to −4.5)	4445 (83.4)	3947 (85.2)	−1.8 (−3.0 to −0.6)	0.003
Brain CT	2674 (70.4)	2248 (71.3)	−1.0 (−5.0 to 3.1)	3557 (67.2)	2926 (68.7)	−1.6 (−3.9 to 0.7)	3735 (65.4)	3152 (63.6)	1.7 (0.2 to 3.3)	0.038
Brain MRI	273 (13.4)	650 (27.8)	−14.4 (−17.7 to −11.1)	973 (30.5)	1475 (42.6)	−12.1 (−14.4 to −9.8)	1656 (39.9)	2043 (51.1)	−11.2 (−12.9 to −9.6)	0.010
Cerebrovascular assessment*	158 (5.3)	571 (25.6)	−20.3 (−22.8 to −17.8)	569 (14.8)	1401 (42.5)	−27.7 (−29.6 to −25.8)	1326 (30.9)	2073 (47.6)	−16.7 (−18.3 to −15.2)	<0.001
Cervical vessel assessment†	91 (5.3)	314 (16.4)	−11.0 (−13.3 to −8.7)	535 (19)	884 (23.1)	−4.1 (−6.1 to −2.2)	1326 (32.5)	1466 (32.3)	0.2 (−1.3 to 1.7)	<0.001
TTE/TEE	407 (11.5)	609 (14.7)	−3.2 (−6.1 to −0.3)	827 (19.5)	1321 (31.8)	−12.3 (−14.4 to −10.3)	1581 (35.3)	1962 (37.8)	−2.4 (−4.0 to −0.9)	<0.001
TTE	404 (11.4)	602 (14.5)	−3.1 (−6.0 to −0.2)	804 (19.1)	1312 (31.6)	−12.5 (−14.6 to −10.5)	1544 (34.7)	1942 (37.3)	−2.6 (−4.2 to −1.1)	<0.001
TEE	9 (0.3)	14 (0.3)	0.0 (−0.4 to 0.5)	99 (1.4)	64 (2.2)	−0.7 (−1.3 to −0.1)	215 (6.8)	129 (2.4)	4.4 (3.7 to 5.1)	<0.001
Holter	181 (7.2)	174 (5.5)	1.7 (−0.5 to 4.0)	477 (8.9)	396 (9.9)	−1.0 (−2.4 to 0.4)	557 (11.2)	496 (11.8)	−0.6 (−1.6 to 0.5)	0.32
24-hour blood pressure monitor	116 (2.5)	83 (3.5)	−1.0 (−2.4 to 0.5)	234 (2.9)	262 (5.4)	−2.5 (−3.4 to −1.6)	374 (8.2)	369 (8.8)	−0.6 (−1.5 to 0.3)	<0.001
Composite score of tests‡	0.18±0.06	0.24±014	−0.06 (−0.07 to −0.05)	0.23±0.13	0.33±0.22	−0.10 (−0.11 to −0.10)	0.32±24	0.37±33	−0.04 (−0.05 to −0.03)	<0.001
Homocysteine	12 (0.9)	24 (0.5)	0.4 (−0.4 to 1.1)	209 (11.3)	646 (20)	−8.7 (−10.3 to −7.1)	1715 (41.2)	2011 (43.1)	−1.9 (−3.5 to −0.3)	<0.001
Glycated haemoglobin	43 (2.1)	186 (4.7)	−2.6 (−4.0 to −1.2)	429 (12.9)	823 (23.3)	−10.4 (−12.1 to −8.6)	1375 (30.8)	1775 (36.5)	−5.7 (−7.2 to −4.2)	<0.001
rt-PA use when arrived within 1 day after onset§	18 (0.3)	4 (0.1)	0.2 (−0.2 to 0.6)	1 (0.0)	2 (0.0)	−0.0 (−0.1 to 0.0)	12 (0.2)	27 (0.8)	−0.6 (−0.8 to −0.4)	
Secondary prevention treatments										
Antiplatelets	2462 (72.1)	1889 (55.6)	16.6 (12.6 to 20.5)	3548 (75.2)	3130 (72.7)	2.4 (0.4 to 4.5)	4130 (75.1)	3764 (77.8)	−2.7 (−4.1 to −1.4)	<0.001
Aspirin	2384 (70.3)	1796 (51.8)	18.5 (14.5 to 22.5)	3442 (70.4)	2872 (63)	7.4 (5.2 to 9.6)	3750 (67.8)	3236 (64.9)	2.9 (1.4 to 4.4)	<0.001
Clopidogrel	12 (0.6)	55 (2.7)	−2.1 (−3.0 to −1.2)	218 (9)	454 (14.9)	−5.9 (−7.4 to −4.4)	1012 (21.9)	1336 (32.2)	−10.3 (−11.7 to −8.9)	0.50
Aspirin plus clopidogrel	11 (0.6)	29 (1.3)	−0.7 (−1.4 to 0.1)	127 (4.5)	205 (5.5)	−1.1 (−2.1 to −0.1)	649 (14.7)	812 (19.3)	−4.6 (−5.7 to −3.4)	0.70
Anticoagulant for AF	3 (2.1)	18 (13)	−10.9 (−18.1 to −3.8)	12 (5.4)	19 (9.0)	−3.6 (−8.8 to 1.6)	18 (6.7)	41 (15.0)	−8.2 (−12.3 to -4.1)	0.45
Antihypertensive drugs	1891 (70.1)	1726 (72.3)	−2.2 (−6.8 to 2.3)	2650 (69.1)	2342 (66.2)	2.8 (0.3 to 5.4)	2616 (62.5)	2378 (64.5)	−2.0 (−3.8 to −0.2)	0.004
Hypoglycaemic drugs	395 (68.2)	567 (66.1)	2.1 (−7.5 to 11.6)	653 (74.9)	794 (73.7)	1.2 (−3.2 to 5.6)	776 (71.0)	938 (68.6)	2.4 (−0.6 to 5.4)	0.14
Statins	412 (11.9)	432 (14.6)	−2.7 (−5.5 to 0.2)	1848 (45.6)	1668 (40.3)	5.2 (2.8 to 7.6)	3404 (68.9)	3161 (70.7)	−1.8 (−3.3 to −0.3)	0.003
Composite score of secondary prevention treatments¶	0.50±12	0.45±0.23	0.05 (0.03 to 0.06)	0.62±0.23	0.59±0.36	0.03 (0.02 to 0.04)	0.68±0.36	0.70±0.49	−0.02 (−0.03 to −0.00)	0.020
Traditional Chinese medicine	2954 (79.4)	2488 (71.3)	8.2 (4.6 to 11.8)	4383 (85.3)	3788 (85.4)	−0.1 (−1.8 to 1.6)	4799 (84.0)	3808 (82.1)	1.9 (0.7 to 3.1)	0.022
Intervention										
Dysphagia screening	48 (0.9)	132 (4.2)	−3.3 (−4.4 to −2.2)	218 (7.3)	254 (6.5)	0.8 (−0.4 to 2.1)	391 (14.5)	292 (7.1)	7.4 (6.4 to 8.4)	<0.001
Rehabilitation assessment	153 (5.7)	211 (8.3)	−2.5 (−4.7 to −0.4)	336 (6.9)	320 (8.2)	−1.2 (−2.5 to 0.1)	358 (6.9)	489 (9.7)	−2.8 (−3.7 to −1.9)	0.41

Data are presented by weighted mean along with SD (mean±SD) or median along with IQR (median (IQR)) for continuous variables and unweighted frequency along with weighted % (95% CI) for categorical variables. A total of 1374 patients (466, 488 and 420 patients in 2005, 2010, and 2015, respectively) with length of stay ≤1 day were excluded from this analysis first because of inadequate time to be given relative tests, treatments or interventions.

*Includes brain CT angiography, magnetic resonance angiography, transcranial Doppler and/or digital subtraction angiography.

†Included cervical CT angiography, magnetic resonance angiography, carotid ultrasound and/or digital subtraction angiography.

‡Composite score of tests was derived from brain CT/MRI, cerebrovascular assessment, cervical vessel assessment, TTE/TEE, Holter and 24-hour blood pressure monitor.

§For most patients, the onset time and admission time to hospitals were not documented, and we could only calculate the time intervals by day.

¶Composite score of secondary prevention treatments was derived from in-hospital antiplatelets, anticoagulant for AF, antihypertensive drugs, hypoglycaemic drugs and statins therapy.

AF, atrial fibrillation; rt-PA, recombinant tissue plasminogen activator; TEE, transoesophageal echocardiography; TTE, transthoracic echocardiography.

**Table 5:** In-hospital outcomes of all patients stratified by years and urban/rural status

	2005			2010			2015			P for interaction
	Rural (N=3986)	Urban (N=3508)	Difference % (95% CI)	Rural (N=5429)	Urban (N=4560)	Difference % (95% CI)	Rural (N=5867)	Urban (N=4927)	Difference % (95% CI)	
In-hospital outcomes										
In-hospital mortality*	48 (1.0)	85 (2.1)	−1.1 (−2.0 to −0.1)	26 (0.3)	48 (0.9)	−0.5 (−0.9 to −0.2)	18 (0.3)	49 (0.9)	−0.7 (−0.9 to −0.4)	0.32
DAMA†	662 (17.2)	543 (14.5)	2.7 (−0.7 to 6.2)	652 (13.4)	602 (11.0)	2.5 (0.7 to 4.2)	658 (11.8)	411 (6.7)	5.0 (4.0 to 6.0)	<0.001
In-hospital mortality or DAMA‡	710 (15.7)	628 (13.3)	2.4 (−0.6 to 5.4)	678 (11.0)	650 (9.5)	1.5 (0.1 to 3.0)	676 (11.4)	460 (6.8)	4.6 (3.6 to 5.5)	<0.001
Comorbidities										
Pneumonia	341 (9.7)	359 (9.8)	−0.1 (−2.5 to 2.4)	504 (9.1)	495 (9.6)	−0.5 (−1.8 to 0.9)	627 (10.1)	577 (10.6)	−0.5 (−1.4 to 0.5)	0.92
Heart failure	158 (3.0)	165 (3.5)	−0.5 (−2.0 to 1.0)	229 (4.3)	253 (3.7)	0.6 (−0.3 to 1.6)	343 (6.0)	365 (5.2)	0.8 (0.1 to 1.5)	0.35
Cerebral haemorrhage	46 (1.1)	69 (2.1)	−1.1 (−2.0 to −0.1)	46 (0.7)	56 (1.0)	−0.2 (−0.6 to 0.2)	89 (1.4)	79 (1.4)	−0.1 (−0.4 to 0.3)	0.034
Symptomatic seizure	45 (1.1)	45 (1.4)	−0.3 (−1.2 to 0.6)	55 (1.1)	59 (1.6)	−0.5 (−1.0 to 0.0)	39 (0.9)	59 (1.0)	−0.1 (−0.4 to 0.2)	0.57
Depression	2 (0.1)	10 (0.6)	−0.5 (−0.9 to −0.1)	16 (0.7)	15 (0.3)	0.5 (0.1 to 0.8)	15 (0.4)	32 (0.7)	−0.3 (−0.5 to −0.1)	0.22
Deep vein thrombosis	5 (0.2)	10 (0.5)	−0.3 (−0.7 to 0.1)	5 (0.1)	10 (0.2)	−0.2 (−0.3 to 0.0)	22 (0.5)	27 (0.3)	0.2 (−0.0 to 0.4)	0.009
Renal dysfunction	61 (1.8)	64 (1.7)	0.1 (−1.0 to 1.2)	108 (2.2)	103 (2.5)	−0.3 (−1.0 to 0.4)	187 (3.2)	201 (3.7)	−0.5 (−1.1 to 0.1)	0.60
Liver dysfunction	53 (1.4)	63 (1.5)	−0.1 (−1.1 to 0.9)	98 (2.7)	122 (2.1)	0.5 (−0.2 to 1.3)	248 (5.7)	276 (4.5)	1.2 (0.5 to 2.0)	0.41
Length of stay less than 10 days*	2283 (56.4)	1322 (33.6)	22.8 (18.7 to 27.0)	2889 (50.2)	1654 (35.0)	15.2 (12.8 to 17.5)	3267 (55.5)	2063 (39.3)	16.2 (14.7 to 17.8)	0.12

Data are presented by weighted median along with IQR (median (IQR)) for continuous variables and unweighted frequency along with weighted % (95% CI) for categorical variables.

*A total of 619 patients (170, 193, and 256 in 2005, 2010, and 2015, respectively) who were transferred out to another hospital were all excluded from this analysis.

†A total of 4058 patients (1241, 1732, and 1084 in 2005, 2010, and 2015, respectively), who died during hospitalisation, were transferred to another hospital or rehabilitation centre or had no documentation of a detailed destination were all excluded from this analysis.

‡Seven hundred fifty-five patients (202, 232, and 321 in 2005, 2010, and 2015, respectively) who were transferred out to another hospital or a rehabilitation centre were all excluded from this analysis.

DAMA, discharge against medical advice.

## Discussion

Using data from a nationally representative sample of two-stage random sampling hospitals and patients for ischaemic stroke in 2005, 2010 and 2015, we found improvements in hospital admission and guideline-recommended management or therapies. Temporal improvement in DAMA and improvements in in-hospital mortality and the composite outcome of in-hospital mortality or DAMA were observed. Disparities between rural and urban hospitals generally decreased but still existed.

Certain temporal trends for ischaemic strokes, such as the growth in the number of hospital admissions and in the prevalence of risk factors, are similar to those previously observed in the USA and the field of ST-segment elevation myocardial infarction in the China PEACE Study.^12 14 16 21–23^ This may be due to several factors. First, the prevalence of cerebrovascular risk factors, including hypertension, diabetes mellitus and dyslipidaemia, is growing, which may be due to lifestyle changes. This has led to a rapid growth in stroke incidence in China and a greater need for an increased rate of access to healthcare utilisation, including hospitalisation, as well as improved health education.^2 3 9 10 24 25^ Corresponding to the increased need is the 118% increase in the number of hospital beds between 2005 and 2015.^26^ The rise in the number of admissions may be due to reimbursement policies that currently favour inpatient treatment, which comprises a bulk of services covered under the premium.^27^ It may also be attributed to improved ability and more chances to access high-quality healthcare over time. Furthermore, the healthcare system in China lacks a hierarchical structure, with a focus on treating acute diseases in hospitals while paying much less attention to long-term management or community-based primary care for controlling risk factors. Turning this inverted pyramid-like structure of the healthcare system and paying more attention to primary care to control risk factors may reduce stroke morbidity and hospitalisation.^28^

Our study showed that the overall physical status of the patients, indicated by coma, limb weakness, aphasia or dysarthria, systolic blood pressure, blood lipid or kidney function, improved over time. This may suggest a growing common sense of risk factor management and an improving health awareness to seek medical care when symptoms are relatively mild.^10^ Meanwhile, the quality of stroke care as measured by performance indicators of diagnostic tests, acute therapy and secondary prevention treatments has improved during the past decade. For example, the use of the Holter test, a routine tool to detect atrial fibrillation after stroke,^29^ has increased over time, although it remained low at only 11.6% in 2015 in our study. This may explain the lower prevalence of comorbid atrial fibrillation in our study (5.6% in 2015) than in the US population (17.1%).^16^

The rate of rt-PA utilisation increased sevenfold over the past decade but was still far below the utilisation rate in the USA and Europe.^30^ This was mainly due to the unorganised and underutilisation of the emergency stroke care system that often leads to long prehospital and in-hospital delays,^31^ as well as the lack of coverage for the cost of alteplase by health insurance plans.^32^ With the appeal of stroke experts, a large-scale construction of stroke centres was launched in 2015,^33^ and the rate of rt-PA utilisation increased by 60% from 2015 to 2019 among these stroke centres.^9^

Using combined aspirin and clopidogrel increased from 1.2% in 2005 to 18.0% in 2015, which may be attributed to the findings of the Clopidogrel in High-Risk Patients with Acute Nondisabling Cerebrovascular Events trial published in 2013 and the updated Chinese stroke guideline in 2014.^17 34^ The use of anticoagulants for atrial fibrillation increased over time, but the use of hypoglycaemic drugs for diabetes after stroke remained 69.5% in 2015. Furthermore, the prescription of antihypertensives decreased over time in our study and was only 64.9% in 2015, compared with 72.4% in 2005. These results are consistent with findings from the China National Stroke Registries.^11^ They indicate that the gaps and obstacles to ideal stroke care persist, likely due to balancing the benefits of treatment with risks and financial considerations.^35^

Encouragingly, our results also showed that DAMA between 2005 and 2015 decreased over time, and the in-hospital mortality and the composite outcome of in-hospital mortality or DAMA improved in 2010 or 2015, while the temporal trend was not significant. The non-significant temporal trends of in-hospital mortality and the composite outcome of in-hospital mortality or DAMA cannot exclude the possibility of meaningful improvements because a temporal trend is a too strong hypothesis that cannot be proven if increased rates were different among the intervals between 2005, 2010 and 2015. In addition, a previous study extracting data from medical records among tertiary hospitals also found that the in-hospital mortality for ischaemic stroke decreased from 2.48% in 2007 to 1.47% in 2010.^36^ The ‘Get With The Guidelines-Stroke’ also demonstrated that in-hospital mortality in the USA decreased over time.^37^ Major factors that likely contributed to reducing stroke mortality in China include the contemporary national healthcare reform plan,^8^ improved healthcare coverage,^38^ new treatment options and modern medical technology.^39^ The in-hospital mortality reported in our study was much lower than other studies from Western countries. One possible reason is that many patients withdraw from treatment at terminal status, which could be attributed to affordability or culture.^35 40^ Therefore, we reported the composite outcome of in-hospital mortality or DAMA, which would reflect a unique discharge pattern in China.

Unlike the China PEACE Study,^41^ rural–urban disparities were only observed for diabetes, not hypertension, dyslipidaemia and a number of risk factors in our study. Rural–urban differences in diagnostic tests, including brain MRI, cerebrovascular assessment and preventive drugs, including clopidogrel and anticoagulant, for atrial fibrillation remain, and more resources and attention should be given to rural hospitals. No significant discrepancies between rural and urban areas were found for DAMA and the composite outcome of in-hospital mortality or DAMA; however, the disparities increased from 2005 to 2015, indicating slower improvement and transition in rural hospitals. The differences in study hospitals, study population and study year may contribute to the differences in urban–rural disparities drawn from these two studies.

To the best of our knowledge, China PROGRESS is the largest study to date of national trends in ischaemic strokes in China with a rigorous random sampling framework of a hospital-based cohort. It placed a strong emphasis on data quality in case ascertainment and data abstraction through the double-blinded data reading and data entry system. Due to the retrospective nature of variable extraction, the low error rate attributed to this system is key to the validity of our findings. Our study sample included hospitals with diverse characteristics in terms of stroke facilities, capacity and quality of care, which allowed us to generate findings that are nationally representative and can be leveraged for quality improvement in a country with great variability in these characteristics across regions and hospitals.^3 39 42^ However, there are several limitations in this study. First, since patients with ischaemic stroke were sampled according to the principal diagnosis of ICD-10 codes on the front page of medical records in this study, the accuracy of this diagnosis could not be determined by data entry from non-stroke professionals. To determine the relatively accurate diagnosis of the index event for hospitalisation, 1400 (5%) records were randomly sampled from the whole population, and two experienced stroke neurologists were invited to make a final confirmation of the diagnosis. Then, 939 (67.1%) confirmed as novel ischaemic stroke, 450 (32%) confirmed as undetermined diagnosis due to inadequate information and 11 (0.8%) identified as misdiagnosed. Second, definitions of disorders and completeness of documentation may vary over time and across hospitals, and it is unclear whether patients were selected from stroke units or other parts of the departments of neurology at participating hospitals. Finally, data on treatments and outcomes after discharge were not collected.

## Conclusions

In conclusion, improvements in hospital admission, stroke management and outcomes in China were observed from 2005 to 2015. However, disparities between rural and urban hospitals in in-hospital management and outcomes persist. Rigorous and systematic quality evaluation and sophisticated incentives for high-value performance are needed to further optimise the quality of care. These findings show that there are many opportunities for policymakers, healthcare systems and health professionals to tailor strategies for resource allocation and disease management in the coming decades.

## Data Availability

Data are available upon reasonable request. Data are available upon reasonable request to the corresponding author.
