# Supplementary material for: Temporal trends and rural–urban disparities in cerebrovascular risk factors, in-hospital management and outcomes in ischaemic strokes in China from 2005 to 2015: a nationwide serial cross-sectional survey
Source: Stroke Vasc Neurol. 2022 Aug 19;8(1):34–50. doi: 10.1136/svn-2022-001552 (PMC9985802; doi:10.1136/svn-2022-001552)
Supplement: Supplementary data [file svn-2022-001552supp001.pdf]

SUPPLEMENTAL MATERIAL

Contents

eTable 1. Definition of in-hospital outcomes, comorbidities, and costs

eTable 2. Specifications of guideline-recommended performance indicators for Ischemic Strokes

eTable 3. The number of observations with missing data for covariates.

eTable 4. Top 10 traditional Chinese medicines used each year

eTable 5. Demographic characteristics of patients stratified by urban/rural and years.

List 1. Participating hospitals of the China-PROGRESS Study

Wang C-J, *et al. Stroke Vasc Neurol* 2023; 8:e001552. doi: 10.1136/svn-2022-001552

eTable 1. Definition of in-hospital outcomes, comorbidities, and costs.

**In-hospital outcomes**	**Definition**
In-hospital mortality[a]	All of the deaths that occur during the hospitalization, regardless of the cause.
Discharge against medical advice (DAMA)[b]	A situation in which a patient chooses to leave the hospital before the managing physician recommends discharge.
In-hospital mortality or DAMA[c]	A situation in which a patient dies regardless of the cause or discharges against medical advice.
Length of stay (LOS)	The length of an inpatient episode of care, calculated from the day of admission to the day of discharge, and based on the number of nights spent in hospital
Pneumonia	Pneumonia was recorded at discharge, regardless of pre-existing or acquired in-hospital.
Heart failure	Heart failure recorded at discharge, regardless of pre-existing or acquired in-hospital.
Cerebral hemorrhage	Cerebral hemorrhage was recorded at discharge but not at admission.
Symptomatic seizure	Seizures occurred after the ischemic stroke event and were recorded at discharge.
Depression	Depression occurred after the ischemic stroke event and was recorded at discharge.
Deep vein thrombosis	Deep vein thrombosis occurred after the ischemic stroke event and was recorded at discharge.
Renal dysfunction	Renal dysfunction occurred after the ischemic stroke event and was recorded at discharge.
Liver dysfunction	Liver dysfunction occurred after the ischemic stroke event and was recorded at discharge.
Total costs	All costs occurred during the hospitalization, which were recorded on the first page of medical records.
Drug costs	Only drug costs (containing traditional Chinese medicine) occurred during the hospitalization, which were recorded on the first page of medical records.

[a] Patients who were transferred to another hospital were excluded from the analysis of in-hospital mortality because there was very limited time to capture in-hospital death.

[b] Patients who were died during hospitalization, transferred to another hospital, a rehabilitation center, or no documentation of detailed destination were all excluded from the analysis of DAMA for eligibility.

[c] Patients who were transferred to another hospital or a rehabilitation center were excluded from the analysis of the composite outcome of In-hospital mortality or DAMA.

eTable 2. Specifications of performance indicators of ischemic Strokes

**stroke/transient ischemic attack care**	**Performance indicator definitions for eligible patients**
Diagnostic tests	Diagnostic testing for stroke etiology includes Brain CT/MRI, Cerebrovascular assessment*, Cervical vessels assessment†, Holter, and 24-hour blood pressure monitor
Antiplatelet	Antiplatelet therapy prescribed during the hospitalization, including aspirin, clopidogrel, or aspirin plus clopidogrel
Anticoagulation for Atrial Fibrillation	Anticoagulation prescribed for patients with atrial fibrillation documented during the hospitalization, including warfarin, heparin, low-molecular-weight heparin, or novel oral anticoagulants (NOACs).
Antihypertension for hypertension disease	Antihypertension medication prescribed for patients with a history of hypertension disease or hypertension disease documented during the hospitalization
Hypoglycemic therapy for diabetes mellitus	Hypoglycemic medication prescribed for patients with a history of diabetes mellitus or diabetes mellitus documented during the hospitalization
Statin	Statin medications prescribed for patients with ischemic cerebrovascular diseases during the hospitalization
Traditional Chinese medication	Traditional Chinese medication prescribed for patients with ischemic stroke during the hospitalization
Dysphagia Screening	Dysphagia screening prior to any oral intake
Rehabilitation assessment	Rehabilitation assessment for patients with ischemic stroke during the hospitalization
Composite score of tests	Defined as the total number of tests or treatments performed among eligible patients divided by the total number of possible tests or treatments among eligible patients.<br>Composite score of tests was derived from brain CT/MRI, cerebrovascular assessment, cervical vessels assessment, TTE/TEE, holter, and 24-hour blood pressure monitor.
Composite score of secondary prevention treatments	Derived from in-hospital antiplatelets, anticoagulants for atrial fibrillation, antihypertensive drugs, hypoglycemic drugs, and statins therapy.

Eligible patients are those without any medical contraindications (eg, treatment intolerance, excessive risk of adverse reaction, patient/family refusal, or terminal illness/comfort care only) documented as reasons for nontreatment for each of the applicable measures. Also excludes patients who were transferred in or whose lengths of stay in hospital did not exceed 24 hours.

* Included brain CT angiography, magnetic resonance angiography, transcranial doppler and/or digital subtraction angiography.

† Included cervical CT angiography, magnetic resonance angiography, carotid ultrasound and/or digital subtraction angiography.

eTable 3. The number of observations with missing data for covariates.

Variables	No. with missing data/Total (%)
Age	35/28277 (0.1)
Smoking history	829/28277 (2.9)
History of stroke	831/28277 (2.9)

eTable 4. Top 10 traditional Chinese medicines used each year.

No.	2005 (N=7494)			2010 (n=9989)			2015 (n=10794)		
	Name of the TCMs	中文名称	Unweighted n (weighted %)	Name of the TCMs	中文名称	Unweighted n (weighted %)	Name of the TCMs	中文名称	Unweighted n (weighted %)
1	Ginko Biloba Extract	银杏叶提取物	1258 (16.5)	Ginko Biloba Extract	银杏叶提取物	1911 (20.4)	Ginko Biloba Extract	银杏叶提取物	1544 (17.6)
2	Sanqi Panax Notoginseng for Injection	血塞通	1391 (14.9)	Injection of Ginkgo biloba extract	舒血宁	1454 (16.7)	Shuxuetong injection[a]	疏血通	1371 (17.1)
3	Puerarin	葛根素	803 (10.1)	Shuxuetong injection [a]	疏血通	965 (14.1)	Injection of Ginkgo biloba extract	舒血宁	980 (13.6)
4	Tongxinluo Capsule[b]	通心络	477 (8.0)	Xueshuantong Injection[c]	血栓通	1387 (12.0)	Xueshuantong Injection[c]	血栓通	1629 (12.9)
5	Xueshuantong Injection[c]	血栓通	678 (7.9)	Sanqi Panax Notoginseng for Injection	血塞通	1573 (11.2)	Sanqi Panax Notoginseng for Injection	血塞通	1668 (11.2)
6	Injection of Ginkgo biloba extract	舒血宁	723 (7.6)	Ginkgo Leaf Extract and Dipyridamole Injection	银杏达莫	1167 (8.0)	Salvia ligustrazine	丹参川芎嗪	883 (9.8)
7	Ginkgo Leaf Extract and Dipyridamole Injection	银杏达莫	360 (6.2)	Naoxintong capsule[d]	脑心通	778 (7.4)	Danhong injection [e]	丹红	1027 (9.3)
8	Breviscapine	灯盏花素	512 (6.0)	Danhong injection [f]	丹红	729 (6.7)	Xingnaojing injection[f]	醒脑静	902 (8.2)
9	Xingnaojing injection[f]	醒脑静	306 (4.3)	Xingnaojing injection[f]	醒脑静	735 (6.0)	Ginkgo Leaf Extract and Dipyridamole Injection	银杏达莫	686 (5.0)
10	Flos Carthami	红花	298 (4.1)	Breviscapine	灯盏花素	643 (5.8)	Naoxintong capsule[d]	脑心通	751 (4.8)

[a] The main component of Shuxuetong injection is total saponins of Panax notoginseng

[b] The main components of Tongxinluo capsule are Ginseng, Hirudo, Scorpio, Radix Paeoniae Rubra, Cicada Slough, Ground Beetle, Scolopendra, Lignum Santali Albi, Lignum Dalbergiae Odoriferae, Frankincense, Semen Ziziphi Spinosae, Borneolum Syntheticum, et al.

[c]The main component of Xueshuantong Injection is total saponins of Panax notoginseng.

[d] The main components of Naoxintong capsule are Radix Astragali, Radix Paeoniae Rubra, Radix Salviae Miltiorrhizae, Radix Angelicae Sinensis, Rhizoma Chuanxiong, Semen Persicae, Flos Carthami, Frankincense, Myrrh, Caulis Spatholobi, Two-Toothed Achyranthes, Ramulus Cinnamomi, Ramulus Mori, Pheretima, Scorpio and Hirudo.

[e] The main components of Danhong injection are Radix Salviae Miltiorrhizae and Flos Carthami.

[f] The main components of Xingnaojing injection are Artifitial Moschus, Radix Curcumae, Borneolum Syntheticum, and Fructus Gardeniae.

eTable 5. Demographic characteristics of patients stratified by urban/rural and years.

	2005			2010			2015		
	**Rural (N=3986)**	**Urban (N=3508)**	**Differece (95% CI)**	**Rural (N=5429)**	**Urban (N=4560)**	**Differece (95% CI)**	**Rural (N=5867)**	**Urban (N=4927)**	**Differece (95% CI)**
**Demographic**									
Age	68.0 (58.0–75.0)	67.0 (56.0–74.0)	0.9 (0.3 to 1.4)	68.0 (60.0–76.0)	68.0 (58.0–75.0)	0.0 (0.0 to 0.1)	68.0 (60.0–76.0)	67.0 (58.0–76.0)	1.0 (1.0 to 2.0)
Female	1602 (39.5)	1399 (38.4)	1.1 (-3.0 to 5.1)	2238 (42.1)	1831 (39.3)	2.7 (0.4 to 5.0)	2517 (44.3)	1999 (37.4)	6.9 (5.4 to 8.5)
Cerebrovascular risk factors									
Hypertension	2820 (73.1)	2490 (69.6)	3.5 (-0.3 to 7.2)	3966 (76.1)	3508 (76.9)	-0.8 (-2.8 to 1.2)	4257 (73.1)	3704 (75.7)	-2.6 (-4.0 to -1.3)
Diabetes	613 (16.1)	853 (24.6)	-8.5 (-11.7 to -5.3)	934 (20.2)	1138 (27.4)	-7.2 (-9.2 to -5.3)	1126(21.2)	1419 (31.3)	-10.0 (-11.4 to -8.7)
Dyslipidemia	267 (7.4)	300 (7.4)	-0.0 (-2.2 to 2.2)	601 (14.4)	727 (14.8)	-0.3 (-1.9 to 1.3)	1025 (20.6)	1084 (24.7)	-4.1 (-5.4 to -2.8)
Atrial fibrillation	271 (7.3)	243 (6.3)	0.9 (-1.2 to 3.1)	299 (5.5)	283 (4.9)	0.6 (-0.5 to 1.6)	335 (5.0)	361 (6.0)	-1.0 (-1.7 to -0.3)
Current smoker	519 (15.7)	505 (13.2)	2.5 (-0.6 to 5.6)	770 (14.2)	780 (19)	-4.9 (-6.6 to -3.1)	755 (13.7)	826 (22.2)	-8.5 (-9.7 to -7.3)
≥1 Number of risk factors	3233 (83.6)	2939 (82.9)	0.7 (-2.4 to 3.8)	4624 (88.7)	4095 (90.2)	-1.5 (-3.0 to -0.1)	5200 (90.4)	4580 (93.9)	-3.6 (-4.4 to -2.7)
**Medical history**									
Stroke	624 (15.9)	727 (23)	-7.1 (-10.4 to -3.9)	1117 (22.2)	1049 (23.6)	-1.3 (-3.4 to 0.7)	1385 (25)	1353 (31.3)	-6.3 (-7.7 to -4.9)
Coronary heart disease	710 (21.4)	736 (20.8)	0.5 (-2.9 to 3.9)	969 (23.1)	1040 (23.8)	-0.7 (-2.6 to 1.3)	1284 (27.2)	1286 (26.9)	0.3 (-1.1 to 1.7)
Myocardial infarction	50 (1.4)	62 (1.7)	-0.3 (-1.3 to 0.7)	57 (1.1)	74 (1.5)	-0.4 (-0.9 to 0.1)	76 (1.3)	99 (2.1)	-0.8 (-1.2 to -0.4)
**Self-payment Insurance**	2015 (53.8)	1380 (34.4)	19.3 (15.2 to 23.4)	1005 (19.5)	578 (14.1)	5.4 (3.6 to 7.1)	411 (6.0)	503 (9.4)	-3.4 (-4.2 to -2.6)
**Clinical characteristics**									
Time from symptom onset to admission within 7d	3450 (86.3)	2899 (81.6)	4.7 (1.7 to 7.6)	4475 (83.2)	3690 (81.2)	2.0 (0.3 to 3.8)	4800 (82.6)	3898 (78.5)	4.2 (2.9 to 5.4)
Coma	119 (3.1)	95 (2.0)	1.1 (-0.3 to 2.5)	89 (1.8)	86 (1.6)	0.2 (-0.4 to 0.8)	67 (1.4)	91 (1.7)	-0.3 (-0.7 to 0.1)
Limb weakness (muscle	1772 (41)	1317(37.5)	3.5 (-0.6 to 7.6)	2094 (34.6)	1539 (34.6)	0.0 (-2.2 to 2.2)	1824(30.3)	1567(34.8)	-4.5 (-5.9 to -3.0)

power 0-4) (No (%))									
Limb weakness (muscle power 0-3) (No (%))	1165 (26.2)	819(23.2)	3.0 (-0.7 to 6.6)	1262 (19.6)	917 (19.4)	0.2 (-1.7 to 2.0)	1007(16.2)	866(18.1)	-1.9 (-3.0 to -0.7)
Aphasia or dysarthria	1072 (29.1)	959 (29.0)	0.0 (-3.9 to 3.9)	1257 (26.4)	1171 (29.9)	-3.5 (-5.7 to -1.4)	1274 (22.4)	1259 (29.8)	-7.4 (-8.8 to -6.0)
Systolic blood pressure (mmHg)	152.6±12.2	149.4±19.7	3.2 (2.0 to 4.4)	149.4±17.6	147.8±26.5	1.6 (0.7 to 2.6)	145.7±23.7	145.6±33.9	0.1 (-0.8 to 0.9)
Cholesterol (mmol/L)	4.7 (4.0–5.5)	4.8 (4.1–5.5)	-0.1 (-0.1 to -0.0)	4.6 (3.9–5.3)	4.8 (4.1–5.6)	0.3 (-0.4 to -0.2)	4.5 (3.9–5.4)	4.6 (3.8–5.3)	0.0 (-0.0 to 0.0)
Low density lipoprotein (mmol/L)	2.8 (2.2–3.3)	2.9 (2.3–3.5)	-0.1 (-0.2 to -0.1)	2.7 (2.2–3.3)	2.8 (2.2–3.4)	-0.1 (-0.2 to -0.0)	2.6 (2.1–3.3)	2.7 (2.1–3.3)	0.0 (-0.0 to 0.0)
Fast glucose (mmol/L)	5.3 (4.7–6.5)	5.6 (4.9–6.9)	-0.3 (-0.3 to -0.2)	5.4 (4.8–6.4)	5.6 (4.9–6.8)	-0.3 (-0.4 to -0.2)	5.4 (4.8–6.6)	5.7 (5.0–7.1)	-0.3 (-0.4 to -0.2)
Uric acid (μmol/)	278.0 (210.0–349.0)	296.0 (232.0–367.0)	-20.8 (-24.6 to -16.9)	278.1 (222.0–347.0)	302.8 (245.0–370.0)	-40.5 (-48.0 to -33.0)	301.0 (244.0–371.0)	312.7 (249.8–379.0)	-8.9 (-13.0 to -4.7)
Alanine transaminase (U/L)	19.0 (13.0–29.0)	18.0 (14.0–27.0)	0.0 (0.0 to 0.2)	18.0 (12.3–26.0)	18.0 (13.0–25.0)	-2.0 (-2.2 to -1.0)	17.0 (12.0–25.9)	18.0 (13.0–26.0)	-1.0 (-1.0 to -0.4)
Estimated glomerular filtration rate (mL/(min·1.73 m^2)	80.5 (64.1–97.7)	83.7 (66.2–100.9)	-2.6 (-3.4 to -1.8)	90.7 (71.5–104.0)	91.5 (72.2–105.1)	-0.0 (-1.9 to 1.9)	95.0 (78.1–106.3)	95.8 (78.8–107.9)	-1.1 (-2.0 to -0.2)

UEBM indicates Urban Employee Basic Medical Insurance; URBM, Urban Resident Basic Medical Insurance; and NRCMS, New Rural Co-operative Medical Insurance Scheme.

Data are presented by weighted mean along with standard deviation (mean±SD) or median along with interquartile range (median [IQR]) for continuous variables, and unweighted frequency along with weighted % [95% CI] for categorical variables.

Wang C-J, *et al. Stroke Vasc Neurol* 2023; 8:e001552. doi: 10.1136/svn-2022-001552

List 1. Participating hospitals of the China-PROGRESS Study

China-PROGRESS retrospective study sites in alphabetical order: Aba People's Hospital, Affiliated Hospital of Guizhou Medical University, Affiliated Hospital of Hainan Medical College, Affiliated Hospital of North China University, Affiliated Hospital of Qingdao University, Alxa League Hospital, Anhui Province Mengcheng County, People's Hospital, Anshan Changda Hospital, Baiquan People's Hospital, Baoding Second Central Hospital, Baotou Fourth Hospital, Beipiao People's Hospital, Beishan Community Hospital of Baishan, Caidian People's Hospital, Changzheng People's Hospital of Shangqiu, Chengwu People's Hospital, Chenxi People's Hospital, Chenzhou First People's Hospital, Chongqing Sixth People's Hospital, Chongren People's Hospital, Dalian Fifth People's Hospital

Danzhou People's Hospital, Dao People's Hospital, Daofu People's Hospital, Daoli People's Hospital, Dashiqiao Central Hospital, Dingyuan People's Hospital, Dongyang People's Hospital, Dunhua Hospital, Fenghuang People's Hospital, Fengshan People's Hospital, First People's Hospital of Yitong Manchu Autonomous County, Fugu Hospital, Fujian Provincial Hospital, Fuling Central Hospital, Fushun Central Hospital, Fuzhou First Hospital, Gannan People's Hospital, General Hospital of Tianjin Medical University, Gongcheng People's Hospital, Gongshan People's Hospital, Guangchang People's Hospital, Guangyuan First People's Hospital, Guangzhou First People's Hospital, Guangzhou Panyu Central Hospital, Guilin People's Hospital, Guiping People's Hospital, Haimen People's Hospital, Haiyan people's hospital, Harbin 242 Hospital, Hegang Mine General Hospital, Helong People's Hospital, Heze Municipal Hospital, Horinger Hospital, Huairen People's Hospital,Huangshan Third People's Hospital, Huayin Central Hospital, Huaying People's Hospital, Huizhou Central People's Hospital, Hunan Provincial People's Hospital, Hunchun People's Hospital, Ji 'an Central People's Hospital, Jiangxi Provincial People's Hospital, Jilin, Hospital of Integrated Traditional Chinese and Western Medicine, Jilin Province People's Hospital, Jinghai Hospital, Jingxi People's Hospital, Jingxing Hospital, Jingyu People's Hospital, Jingzhou Central Hospital,

Jinning People's Hospital, Jiuquan People's Hospital, Jixi People's Hospital, Jize Hospital, Kaifeng Central Hospital, Kaiping Hospital, Kangbao People's Hospital, Keshikesketeng People's Hospital, Kirin People's Hospital, Lanping People's Hospital, Leting Hospital, Liaoyang Central Hospital, Liaoyang Third People's Hospital, Liaoyuan Central Hospital, Liaoyuan Second People's Hospital, Linchuan People's Hospital, Lindian Hospital, Linxiang People's Hospital, Liujiang People's Hospital, Longyan First Hospital, Luancheng People's Hospital, Lucheng People's Hospital, Luchuan People's Hospital, Luxi People's Hospital, Macheng People's Hospital, Menglian People's Hospital, Minxian People's Hospital of Gansu Province, Nanan Hospital, Nanhu Central Hospital of Jiaxing, Nanjing First Hospital, Nantong Third People's Hospital, Nanxun people's hospital, Nanyang Central Hospital, Ningwu People's Hospital, People's Hospital of Boltala Mongolian Autonomous Prefecture, People's Hospital of Jianghua Hao Autonomous County, People's Hospital of Muli Tibetan Autonomous County, People's Hospital of Nanchuan, Chongqing, People's Hospital of Tibet Autonomous Region, People's Hospital of Yuanzhou District of Ningxia, Pianguan People's Hospital, Puding People's Hospital, Qinghai Red Cross Hospital, Qinshui People's Hospital, Qinyang People's Hospital, Qiqihar First Factory Hospital, Qujiang People's Hospital, Quzhou People's Hospital, Rencheng People's Hospital, Rongjiang People's Hospital, Rudong Hospital, Ruyang People's Hospital, Ruzhou First People's Hospital, Second Affiliated Hospital of Harbin Medical University, Shangluo Central Hospital, Shangqiu Fourth People's Hospital, Shaoyang People's Hospital, Shengsi County People's Hospital, Shougang Shuigang General Hospital, Shuangshan Hospital of Anshan, Siziwang Banner People's Hospital, Taizhou Hospital of Zhejiang Province, The Fifth Hospital of Qinghai Province, The First Affiliated Hospital of Hebei North University, The First Affiliated Hospital of Henan University of Science and Technology, The First Affiliated Hospital of Jiamusi University, The First People's Hospital of Xinmi, The Second affiliated Hospital of Hebei North University, The Second Affiliated Hospital of Kunming Medical University, The Second Hospital of Saihan District of Hohhot, The Third People's

Hospital of Xining, Tieling People's Hospital, Tiexi Central Hospital of Shenyang, Tongchuan Mining Bureau Central Hospital, Tongliang People's Hospital, Tongliao First People's Hospital, Ulanqab Central Hospital, Urat Front Banner People's Hospital, Wencheng People's Hospital, Wuchuan People's Hospital, Wuhai People's Hospital, Wuhu First People's Hospital, Wuhu Second People's Hospital, Wuqiang Hospital, Wuyishan Municipal Hospital, Xi 'an First Hospital, Xiangtan People's Hospital, Xigu Hospital of Lanzhou, Xing People's Hospital, Xinghualing Central Hospital, Xingyang People's Hospital, Xinhua Hospital Affiliated to Dalian University, Xinmi Zhongkang Hospital, Xinshao people's hospital, Xiuwu People's Hospital, Xuanhan People's Hospital, Xupu People's Hospital, Yanggao People's Hospital, Yicheng People's Hospital, Yichun Second People's Hospital, Ying People's Hospital, Yongxing People's Hospital, Yongzhou Central Hospital, Yongzhou Third People's Hospital, Yuanling People's Hospital, Yuci People's Hospital, Yueqing People's Hospital, Yuhong People's Hospital, Yuncheng Central Hospital, Yunlong People's Hospital, Yuyao People's Hospital, Zhangjiachuan First People's Hospital, Zhengzhou Central Hhospital, Zhengzhou Fifth People's Hospital, Zhijiang People's Hospital, Zhouning Hospital, Zhuozi Hospital, Zigong Fourth People's Hospital, Zuoyun People's Hospital.

Wang C-J, *et al. Stroke Vasc Neurol* 2023; 8:e001552. doi: 10.1136/svn-2022-001552
